# Posterior Tibial Tendon Tenosynovitis Diagnosed by Point-of-Care Ultrasound

**DOI:** 10.5811/cpcem.2017.6.34430

**Published:** 2017-10-18

**Authors:** Shawna D. Bellew, Kristina M. Colbenson, Venkatesh R. Bellamkonda

**Affiliations:** * Vanderbilt University Medical Center, Department of Emergency Medicine, Nashville, Tennessee; † Mayo Clinic, Department of Emergency Medicine, Rochester, Minnesota; ‡ Mayo Clinic Sports Medicine Center, Mayo Clinic, Rochester, Minnesota

## CASE PRESENTATION

A 48-year-old woman presented with right ankle pain that began while running two days prior. She noted that the ankle hurt with even light touch and the pain was unrelieved with ibuprofen. She denied a history of trauma. She was seen in the emergency department for this condition the day prior with a negative radiograph, but she returned because of increased ongoing pain. On examination, a five-centimeter (cm) area of erythema was found posterior to the medial malleolus and parallel to the posterior tibial tendon (PTT). The diameter, measured with point-of-care ultrasonography (using SonoSite X-Porte with L38xp 10–5 MHz linear transducer; SonoSite, Inc, Bothell, WA) of the long axis of the tendon in this region, was 4.9 millimeter (mm) (reference range, 3.1–4.6 mm)^1^ with anechoic fluid visible in the peritendinous space (Image). The patient received a diagnosis of posterior tibial tendon tenosynovitis with posterior tibial nerve neuralgia. Her prescribed treatment was anti-inflammatory medications and rest. She had complete resolution of her symptoms at eight weeks, at which time she resumed full activity.

## DISCUSSION

The PTT is important for plantar flexion, inversion and supination of the ankle, as well as stabilizing the arch of the foot.^2^,^3^ Thickening of the PTT and peritendinous fluid are ultrasonographic characteristics of PTT tenosynovitis.^1^,^2^ This condition can occur in healthy young athletes from overuse and poor biomechanics caused by microtrauma or systemic inflammatory diseases.^4^,^5^ Tenosynovitis narrowed the functional space within the enclosed tarsal tunnel, leading to posterior tibial nerve compression neuralgia that caused hyperesthesia. Unlike infectious tenosynovitis, inflammatory tenosynovitis often is managed nonoperatively. The mainstay of treatment includes anti-inflammatory medications, activity modification, foot orthosis, and physical therapy to improve stability and inhibit overpronation. Refractory cases may require corticosteroid injections or surgical intervention.^6^

**Educational Merit Capsule**

What do we already know about this clinical entity?

Posterior tibial tendon tenosynovitis is an inflammatory condition affecting healthy young athletes and can be associated with posterior tibial nerve hyperesthesia.

What is the major impact of the image?

This image adds to the growing literature describing the use of ultrasound for evaluation of posterior tibial tendon and helps broaden the differential diagnosis related to ankle pain by emergency department providers.

How might this improve emergency medicine practice?

Ankle pain is a common presentation in EM practice. This image helps to highlight tenosynovitis of the posterior tibial tendon within the differential diagnosis and emphasizes the value of point-of-care ultrasound in establishing the diagnosis.

## Figures and Tables

**Image:**
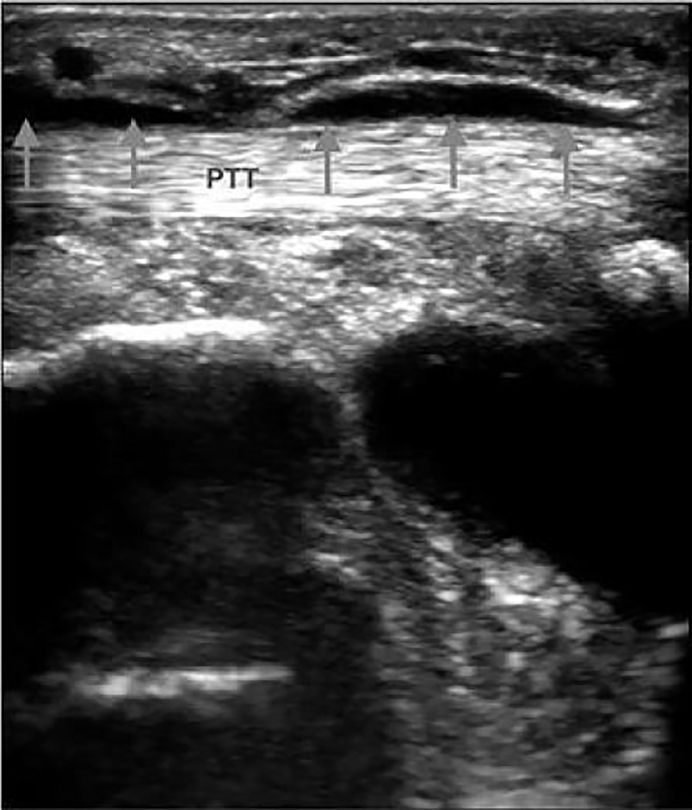
Longitudinal view of posterior tibial tendon (PTT) with anechoic fluid in the peritendinous space (arrows).
